# Supplementary material for: Genome-scale metabolic models for natural and long-term drug-induced viral control in HIV infection
Source: Life Sci Alliance. 2022 May 10;5(9):e202201405. doi: 10.26508/lsa.202201405 (PMC9095731; doi:10.26508/lsa.202201405)
Supplement: Supplementary file 1 [file LSA-2022-01405_TableS1.docx]

**Table S1.** Clinical and demographic characteristics of the patient populations.

|  | PLWH_ART_ | PLWH_EC_ | PLWH_VP_ | HC | P |
| --- | --- | --- | --- | --- | --- |
| N | 19 | 19 | 19 | 19 |  |
| Age, years; median (IQR) | 55 (45-61) | 46 (40-52) | 46 (32-53) | 49 (46-52) | 0.027* |
| Gender, Female; n (%) | 6 (31.6) | 9 (47.4%) | 7 (36.8) | 8 (42.1) | ns** |
| Duration of treatment in years; Median (IQR)^#^ | 17 (7-20) | - | - | - | - |
| Duration of suppressive therapy in years; Median (IQR) ^#^ | 13 (7-17) | - | - | - | - |
| Nadir CD4^+^ T-cell, cells/µl; median (IQR) | 280 (130-420) | 530 (400-680) | 280 (180-350) | - | <0.001* |
| CD4^+^ T-cell, cells/µl; median (IQR) ^#^ | 576 (520-700) | 880 (730-1050) | 300 (180-410) | - | <0.001* |
| CD8^+^ T-cell, cells/µl; median (IQR) ^#^ | 580(450-730) | 780(525-890) | 740(530-910) | - | ns |
| CD4:CD8 Ratio; median (IQR) ^#^ | 1.09 (0.71-1.45) | 1.30 (0.8-1.65) | 0.3 (0.2-0.6) | - | <0.001* |
| Treatment Regimen; n (%) ^#^  ABC/3TC/DRVr  ABC/3TC/DTG  ABC/3TC/EFV  ABC/3TC/NVP  ABC/3TC/RPV  DRV/COB/DTG  RAL/3TC/EFV  TDF/FTC/RPV  TAF/FTC/DTG  TAF/FTC/EFV | 1 (5.25)  3 (15.8)  2 (10.5)  2 (10.5)  3 (15.8)  1 (5.25)  1 (5.25)  3 (15.8)  2 (10.5)  1 (5.25) | - | - | - | - |

*Kruskal-Wallis, **Chi square test, ^#^At sampling
