# Supplementary figures and images for: Genome-scale metabolic models for natural and long-term drug-induced viral control in HIV infection
Source: Life Sci Alliance. 2022 May 10;5(9):e202201405. doi: 10.26508/lsa.202201405 (PMC9095731; doi:10.26508/lsa.202201405)

Replicate 1

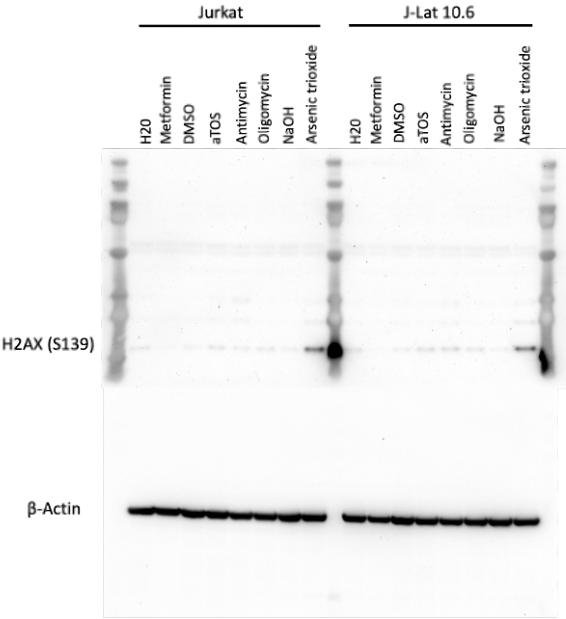

Replicate 2

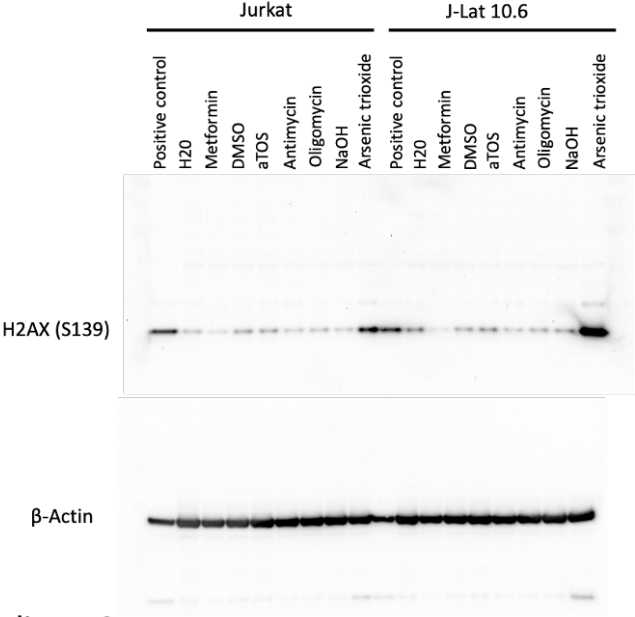

Replicate 3

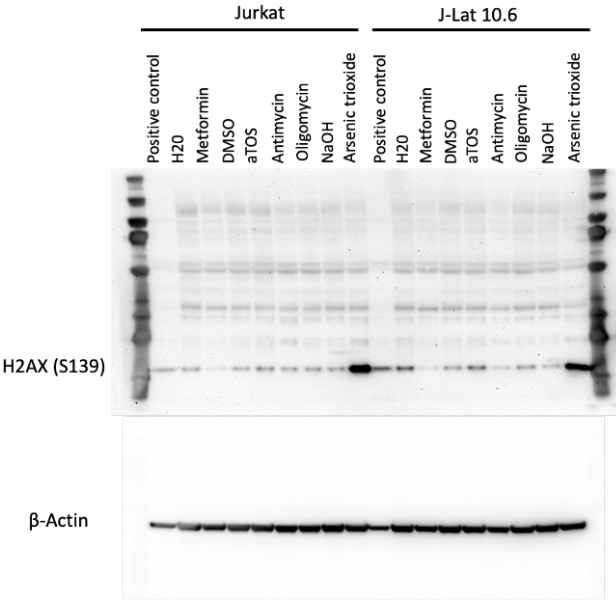

Supplement: Supplementary file 5 [file LSA-2022-01405_SdataF5.pdf]
